# Nosocomial transmission of chickenpox and varicella zoster virus seroprevalence rate amongst healthcare workers in a teaching hospital in China

**DOI:** 10.1186/s12879-019-4222-x

**Published:** 2019-07-05

**Authors:** Jin Yang, Jieling Liu, Fanfan Xing, Haiyan Ye, Guijian Dai, Meiyuan Liu, Simon Kam-Fai Lo, Ricky Wing-Tong Lau, Kelvin Hei-Yeung Chiu, Jasper Fuk-Woo Chan, Kwok-Yung Yuen

**Affiliations:** 1 Department of Clinical Microbiology and Infection Control, The University of Hong Kong – Shenzhen Hospital, Shenzhen, China; 2 State Key Laboratory of Emerging Infectious Diseases, The University of Hong Kong, Pokfulam, Hong Kong, Special Administrative Region of China; 3 Department of Microbiology, Li Ka Shing Faculty of Medicine, The University of Hong Kong, Pokfulam, Hong Kong, Special Administrative Region of China; 4 Carol Yu Centre for Infection, Li Ka Shing Faculty of Medicine, The University of Hong Kong, Pokfulam, Hong Kong, Special Administrative Region of China; 5 The Collaborative Innovation Center for Diagnosis and Treatment of Infectious Diseases, The University of Hong Kong, Pokfulam, Hong Kong, Special Administrative Region of China

**Keywords:** Chickenpox, China, Healthcare worker, Outbreak, Seroprevalence, Varicella zoster virus

## Abstract

**Background:**

Varicella zoster virus (VZV) is a highly contagious herpesvirus with potential for nosocomial transmission. However, the importance of nosocomial chickenpox outbreak in China has often been ignored. With the increasing immunocompromised population in China, a thorough review of issues related to nosocomial transmission and the seroprevalence rate of VZV among healthcare workers is necessary.

**Methods:**

Retrospective case finding for nosocomial transmission of chickenpox was conducted between January 1, 2013 and December 31, 2017. Cases were identified based on clinical features compatible with chickenpox. A cross-sectional study on the seroprevalence rate of VZV among healthcare workers (HCWs) was conducted between January 1, 2014 and December 31, 2017. The serum VZV antibodies of 1804 HCWs were measured by enzyme-linked immunosorbent assay (ELISA). The seroprevalence rate of VZV antibodies, the positive predictive value and negative predictive value of self-reported history of varicella were analyzed. The economic impact associated with nosocomial transmission of VZV was also assessed.

**Results:**

A total of 8 cases of chickenpox were identified in three nosocomial transmissions, including 4 HCWs who were infected nosocomially. The overall seroprevalence rate of VZV was 88.4%, which significantly increased with age (*P* < 0.01). The seroprevalence rates of HCWs with different genders and occupations showed no statistically significant differences. The positive and negative predictive values of a self-reported history of varicella were 80.8 and 10.6% respectively. An estimation of 163.3 person-days of work were lost in each nosocomial transmission and 86.7 infection control unit person-hours were required for each outbreak investigation. The cost of VZV IgG ELISA screening was estimated to be 83 USD per nosocomial transmission.

**Conclusions:**

Nosocomial transmission of VZV occurred repeatedly in the hospital setting. An alarming 11.6% of HCWs were seronegative for VZV, which might increase the risk of nosocomial infection and outbreak for other susceptible co-workers and patients. This is especially important in the setting of a teaching hospital where many immunocompromised patients were managed. Furthermore, the positive predictive value of self-reported varicella on seroprevalence rate in our study was lower than those reported in other countries, therefore serological testing of VZV antibodies with subsequent vaccination for all non-immune HCWs should be considered.

## Background

Varicella zoster virus (VZV) causes two clinically distinct forms of disease: varicella (chickenpox), as a primary infection, and zoster (shingles) due to reactivation of latent VZV [1–3]. The transmission of VZV occurs via inhalation of airborne droplets or direct contact with vesicular fluid from skin lesions. VZV infection is considered an occupational hazard for susceptible healthcare workers (HCWs), as it can spread to other susceptible coworkers and patients [4]. Transmission of VZV among HCWs and patients has been reported [5–7]. Current policy in China for prevention of nosocomial transmission of VZV is prompt recognition of patients with suspected VZV infection with airborne isolation in negative pressure single room if possible, however mandatory VZV IgG testing is still not yet implemented. Thus HCWs with negative history of chickenpox and VZV vaccination should be screened for VZV antibodies. Immune status to VZV can be assessed by enzyme-linked immunosorbent assays (ELISAs), or fluorescent antibody to membrane antigen (FAMA) assays [8]. Seronegative HCWs are considered susceptible to varicella, therefore VZV vaccination is recommended [4].

Primary VZV infection is usually self-limiting in immunocompetent hosts, however, the severity increases with age. Furthermore, as HCWs care for patients in different wards and units, there is a high potential of nosocomial transmission among HCWs and patients if the HCW is infected, especially when the infectious period of VZV starts 2 days before the onset of rash. The situation would be worse if the infected HCW is involved in taking care of pregnant women and immunocompromised individuals (such as patients receiving high dose immunosuppressant or history of transplantation), as primary VZV infection in immunocompromised patients can be fulminant, and it can present as encephalitis, pneumonitis and hepatitis. The mortality rate of primary VZV infection could be up to 7% if left untreated. As a result, nosocomial transmission of VZV should never be overlooked.

As nosocomial transmission of varicella is seldom reported in China, this study aims to report three nosocomial transmissions of chickenpox, and investigate the seroprevalence rate of VZV among healthcare workers in a tertiary teaching hospital in China.

## Methods

### Outbreak investigation and management of nosocomial transmission of chickenpox

Retrospective case finding for nosocomial transmission of chickenpox was conducted, with specific attention being paid to infection control measures and outbreak investigations between January 1, 2013 and December 31, 2017. The clinical definition of varicella is “an illness with acute onset of diffuse (generalized) papulovesicular rash without other apparent cause”. All contacts in the hospital were followed up by the infection control unit staff to determine the number of susceptible contacts (non-immune to varicella) by contact tracing. Susceptible contacts were put under self-medical surveillance and requested to refrain from patient contact from Day 8 after 1st exposure to Day 21 after last exposure. Individuals were considered susceptible if they did not fulfill any one of the following criteria: 1) History of varicella; 2) Completion of varicella vaccine according to recommended schedule; 3) Positive serum VZV IgG. Post-exposure prophylaxis (PEP) for susceptible contacts were administered according to CDC recommendations [2].

### Seroprevalence study of HCWs VZV antibodies

A cross-sectional study on the seroprevalence rate of VZV among healthcare workers (HCWs) was conducted between January 1, 2014 and December 31, 2017 in a tertiary teaching hospital in China, with 1804 hospital staff including doctors, nurses, technicians and non-clinical workers participated in this study. The study was approved by the Ethics Committee of the hospital. Through questionnaire, information including demographic data, history of varicella and vaccination were collected from the participants. Blood samples were collected from each individual and the serum VZV antibodies were measured by enzyme-linked immunosorbent assay (ELISA) using commercial kit (Beier, Beijing, China; based on manufacturer’s data, the positive cutoff was defined as 0.15 if mean of absorbance of negative control < 0.05, while 0.1 + mean of absorbance of negative control if mean of absorbance of negative control ≥0.05, with sensitivity and specificity quoted as 96.1 and 100% respectively).

Statistical analysis was performed using SPSS 20 software. Categorical variables were compared using Chi-square test. A *P*-value of less than 0.05 was considered statistically significant. Positive predictive value (PPV) and negative predictive value (NPV) of a self-reported history of varicella were calculated, with a positive serum VZV IgG considered as the gold standard of immunity against VZV.

### Economic evaluation of nosocomial transmission

In order to assess the economic impact of nosocomial transmission of VZV, two important parameters, person-days of work lost and infection control unit person-hours, were assessed. Person-days of work lost is defined as the total number of days of work loss in clinical staff for both susceptible contacts and infected cases. Infection control unit person-hours is defined as the number of hours required for infection control unit for outbreak investigation and control. The cost of performing VZV IgG ELISA was also calculated based on the price of equipment together with the manpower required to perform the test.

## Results

### Outbreak investigation and control

**Fig. 1:**
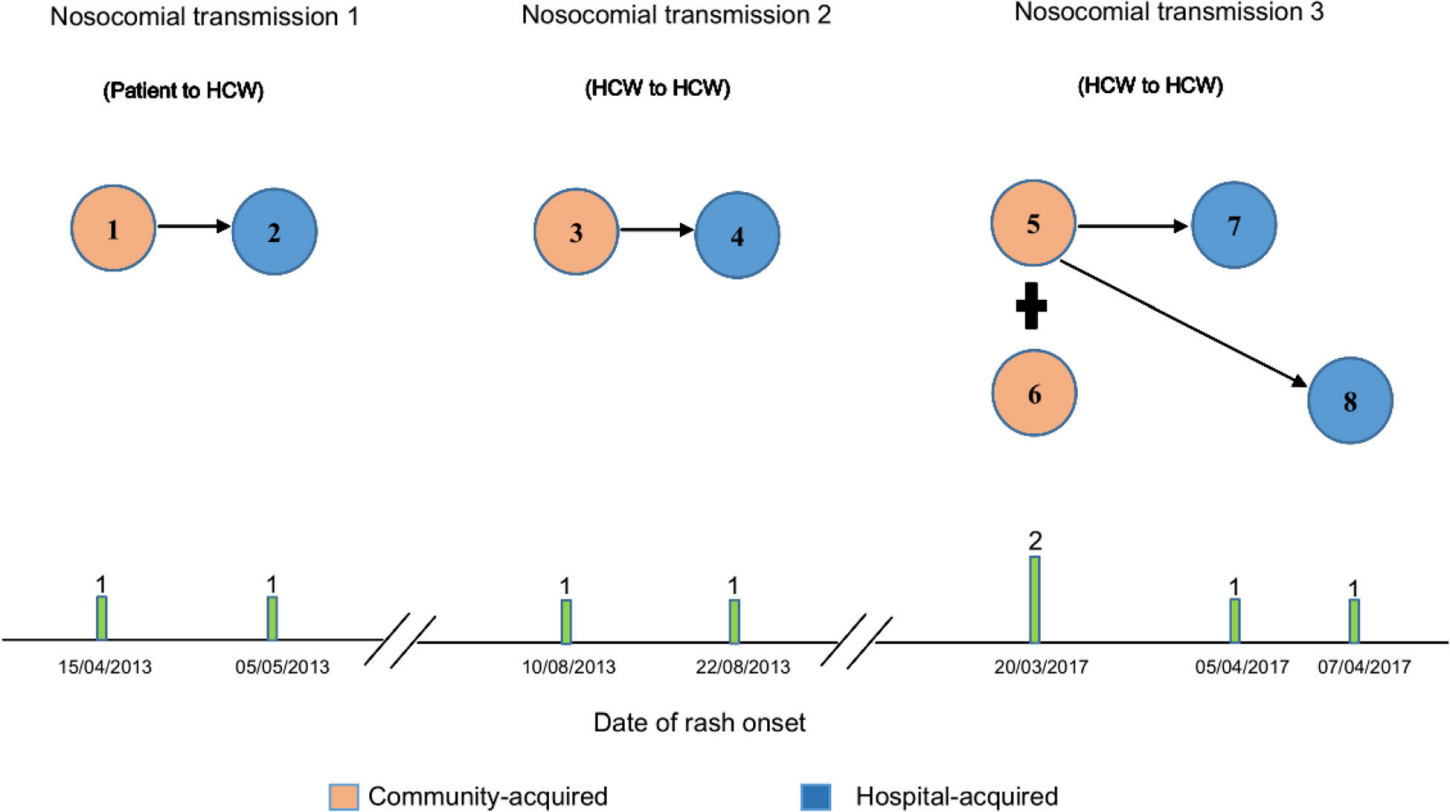
Timeline illustrating epidemic curve and chain of nosocomial transmission of chickenpox in our study, 2013–2017

There were three episodes of nosocomial transmission of varicella involving 8 individuals, in which 4 HCWs (including one doctor, one non-clinical worker and two nursing students) were infected nosocomially. Table 1 shows the characteristics of the 8 cases of varicella, and Fig. 1 shows the epidemic curve and chain of transmission in these episodes.

**Table 1:** Characteristics of 8 chickenpox cases in three nosocomial transmissions, 2013–2017

Event	Case no.	Occupation	Underlying disease or Pregnancy	Date of rash onset	Symptoms			Transmission setting	Total contacts
					fever	generalized vesicular rash	sore throat		
Nosocomial transmission 1									23
	1	NA (Patient)	No	15/04/2013	+	+	–	Community	
	2	Doctor (HCW)	No	05/05/2013	+	+	+	Hospital	
Nosocomial transmission 2									17
	3	Non-clinical staff (HCW)	No	10/08/2013	–	+	–	Community	
	4	Non-clinical staff (HCW)	No	22/08/2013	+	+	+	Hospital	
Nosocomial transmission 3									87
	5	Nursing student (HCW)	No	20/03/2017	+	+	–	Community	
	6	Nursing student (HCW)	No	20/03/2017	+	+	–	Community	
	7	Nursing student (HCW)	No	05/04/2017	+	+	–	Hospital	
	8	Nursing student (HCW)	No	07/04/2017	+	+	–	Hospital

### Nosocomial transmission 1

On May 5, 2013, a paediatrician (Case 2) presented with fever followed by generalized vesicular rash. She recalled contacting a patient (Case 1) with chickenpox on April 18, 2013 in the hospital, and she was susceptible to VZV, i.e., she was neither vaccinated nor previously infected with VZV. She was diagnosed to have varicella and requested self-isolation to prevent ongoing transmission. Contact tracing was initiated by the hospital infection control unit and a total of 23 healthcare worker contacts were identified with advice on prodromal symptoms to ensure they were not exposed to other susceptible HCWs or patients. No further new cases were identified after the above infection control measures. After this incident, serum VZV IgG testing for HCW was introduced by hospital health direction.

### Nosocomial transmission 2

On August 22, 2013, a non-immune operator from the hospital reception center (Case 4) presented with fever, generalized vesicular rash and sore throat. She recalled one of her colleagues (Case 3), who worked with her in the same office, developed chickenpox during August 10–17, 2013. She was subsequently diagnosed to have varicella. The infection control unit was informed of the incident, with 17 healthcare worker contacts identified through contact tracing. Further history taking and laboratory testing suggested 4 of the 17 contacts were susceptible, therefore they all received post-exposure prophylaxis with oral acyclovir. No further new cases were identified after the above infection control measures.

### Nosocomial transmission 3

On March 20, 2017, two nursing students (Case 5 and Case 6) from hospital outsourcing company reported to have fever and generalized vesicular rash. They worked as nursing assistants in the hospital and lived in a 8-person dormitory room. They were diagnosed to have varicella and requested self-isolation. The infection control unit was informed, with 42 healthcare worker contacts identified through contact tracing. Thorough investigation suggested 5 out of 42 contacts were susceptible. All susceptible contacts received VZV vaccination as Post-exposure Prophylaxis (PEP), with recommendation on self-isolation during incubation period provided.

On April 5, 2017 and April 7, 2017, two nursing students (Case 7 and Case 8), who lived with Case 5 in the same dormitory, presented with similar clinical manifestations as Case 5, with VZV DNA detected from the vesicular fluid of skin lesions in both nursing students by PCR (Polymerase Chain Reaction). These two infected individuals were not classified as susceptible contacts during the first round of contact tracing, because both of them volunteered previous history of VZV vaccination. A further 45 healthcare worker contacts were identified through second round of contact tracing, and 4 out of 45 contacts were classified as susceptible individuals, with subsequent post-exposure prophylaxis provided. No further new cases were identified after the above infection control measures.

### Seroprevalence of VZV in HCWs

Of the 1804 HCWs participated, there were 215 (11.9%) male and 1589 (88.1%) female HCWs, with their ages ranging from 17 to 60 years (median age: 27 years). Table 2 shows the demographic characteristics of the participated HCWs. The overall seroprevalence rate of VZV was 88.4%. The seroprevalence rates of HCWs with different genders and occupations showed no statistically significant differences. HCWs aged ≥36 years were found to have the highest seroprevalence rate (92.6%), while HCWs aged ≤25 years were found to have the lowest seroprevalence rate (85.2%).

**Table 2:** Demographic data and VZV serostatus of 1804 healthcare workers

Variable	Total no. (%)	VZV antibodies results		
		Positive no.(%)	Negative no.(%)	*P* value
Gender				0.353
Male	215 (11.9)	186 (86.5)	29 (13.5)	
Female	1589 (88.1)	1409 (88.7)	180 (11.3)	
Age				< 0.01
≤25	809 (44.9)	689 (85.2)	120 (14.8)	
25–35	859 (47.6)	780 (90.8)	79 (9.2)	
≥36	136 (7.5)	126 (92.6)	10 (7.4)	
Occupation				0.382
Physician	153 (8.5)	133 (86.9)	20 (13.1)	
Nurse	1238 (68.6)	1097 (88.6)	141 (11.4)	
Technician	221 (12.3)	190 (86.0)	31 (14.0)	
Non-clinical workers	192 (10.6)	175 (91.1)	17 (8.9)	
Total	1804	1595 (88.4%)	209 (11.6%)	

Table 3 shows the positive and negative predictive values of a self-reported history of varicella, with a positive serum VZV IgG considered as the gold standard of immunity against VZV. Of the 1804 participants, 214 (11.9%) HCWs reported history of varicella, with 173 (80.8%) tested positive and 41 (19.2%) tested negative for serum VZV IgG; while 1590 (88.1%) HCWs reported no history of varicella, with 1422 (89.4%) tested positive and 168 (10.6%) tested negative for serum VZV IgG. The PPV and NPV of a self-reported history of varicella were 80.8 and 10.6% respectively.

**Table 3:** VZV serological test results and history of varicella among 1804 HCWs

History of Varicella	VZV antibody results				
	Total (no.)	Positive no.(%)	Negative no.(%)	PPV	NPV
Positive	214	173 (80.8)	41 (19.2)	80.8	10.6
Negative^a^	1590	1422 (89.4)	168 (10.6)		

^a^including uncertain history

### Economic evaluation of nosocomial transmission

A total of 20 person-days of work were lost by infected clinical staff during the above episodes, based on the fact that immunocompetent persons are unlikely to be infectious after day 5 of rash. Isolating susceptible healthcare worker contacts from duty from days 8 to 21 after last exposure would result in further working hours lost, with 470 person-hours loss for the above three episodes. Therefore, a total of 490 person-hours were lost during the above described three transmissions, with an estimate of 163.3 person-hours being lost per nosocomial transmission.

During the first two transmissions, infection control unit required 40 h per transmission to contact trace, follow up and provide counseling to all involved healthcare workers. As the third transmission involved more clinical staff, infection control unit required 180 h for infection control measures. Therefore, a total of 260 infection control unit person-hours were required for the three transmissions, with 86.7 infection control unit person-hours required per transmission.

The cost of each VZV IgG ELISA commercial kit is 11 RMB (equivalent to 1.59 USD), and manpower for performing each ELISA kit is 2.5RMB (equivalent to 0.36 USD) in our laboratory. With a total of 127 tests performed as a result of nosocomial transmissions, the total cost of VZV IgG ELISA screening is 1714.5 RMB (around 248 USD), with around 83 USD per nosocomial transmission.

## Discussion

Varicella zoster virus (VZV) is a highly contagious herpesvirus with potential for nosocomial transmission, with the infectious period starting 2 days before the onset of rash, and lasting for 5–7 days after the appearance of rash [9–11]. Several studies have demonstrated varicella can be transmitted to susceptible individuals through airborne route [12, 13]. Susceptible individuals can acquire primary infection from contacting patients with dermatomal zoster [12–18]. It is shown in our study that nosocomial transmission is possible.

Nosocomial outbreaks of varicella involving both healthcare workers and patients are reported in other countries [5, 19–22]. Common measures for prevention of varicella transmission in the hospital setting include rapid isolation of patient in negative pressure room together with administration of post-exposure prophylaxis to susceptible individuals in the form of varicella zoster vaccine, varicella zoster immunoglobulin, and oral acyclovir. Majority of the studies showed difficulty in prevention of secondary cases after an index case of varicella was identified in the hospital. Common difficulties encountered include improper isolation facilities and non-specific symptoms during initial presentation, especially when the infectious period of VZV infection starts 2 days before the onset of rash [5]. Furthermore, secondary cases still occurred with post-exposure prophylaxis despite it was given within timeframe [20]. Studies in developing countries such as Indonesia also encountered the problem of lack of supply of varicella zoster immunoglobulin [22]. With the multiple difficulties encountered from previous studies and our experience, routine screening of serum VZV IgG in healthcare workers might be required to reduce the chance of nosocomial transmission in the future.

The seroprevalence rate of VZV in healthcare workers varies according to different geographical areas, with the prevalence of seronegativity ranging from < 5% in the USA, 8.9% in Taiwan, 19% in Saudi Arabia, 26% in India to approximately 50% in Sri Lanka [23, 24]. In our study, the serum VZV antibodies of 1804 HCWs were measured with 88.4% found to be seropositive. The results showed that a considerable proportion (11.6%) of HCWs were still susceptible to varicella, and HCWs can be a potential source of nosocomial transmission, indicating the importance of the screening of serum VZV IgG and vaccination of susceptible HCWs, as exemplified by the three episodes of nosocomial transmissions documented in our hospital in the past 5 years. All the 4 HCWs involved were not screened for serum VZV IgG prior to employment, as a result, they were involved in the three episodes of nosocomial transmission. Nosocomial VZV infection has great economic impact, as laboratory testing, contact tracing and self-isolation of HCWs could represent both an increase in laboratory cost and a decrease in manpower. A reasonable estimate of 163.3 person-days of work were lost as a result of each nosocomial transmission and 86.7 infection control unit person-hours were required during each outbreak investigation and control, together with an estimated laboratory cost of 83 USD per nosocomial transmission, depending on the size of the outbreak.

In our study, the seroprevalence rate of VZV in HCWs significantly increased with age, and this could be explained by accumulated time of exposure to VZV during lifetime in elderly. The seroprevalence rates of HCWs with different genders and occupations showed no statistically significant differences.

In order to investigate whether self-reported history could replace serum VZV IgG screening in HCWs, self-reported history of varicella in HCWs was compared with the serum VZV IgG result to assess the reliability of self-reported history. Our study suggested that a positive history of varicella provided by HCWs is not a good predictor of positive serum VZV IgG, as our study demonstrated a false positive rate of 19.2%. On the other hand, 89.4% of HCWs with a negative history of varicella turned out to be seropositive, suggesting negative self-reported history could not rule out existing immunity towards VZV. Review of literature from different countries suggested a self-reported history of varicella has a positive predictive value of 92.5–99.5% and a negative predictive value of 2.5–14.4% [24–27]. Comparison of the above data suggested that the negative predictive value from our study is similar to that reported in the literature. However, the positive predictive value is only 80.8% in our study. As natural varicella usually occurred during childhood, healthcare workers could only rely on their parents for the history of varicella. One possible postulation for the above discrepancy could be due to the lower level of education in the past in China, as it may be difficult for parents to distinguish between varicella and other febrile exanthematous diseases, leading to lower positive predictive value in this case. Hence, self-reported history of varicella is not a reliable predictor of VZV immunity in our locality, therefore screening of serum VZV IgG for all HCWs is indicated, and susceptible workers should be vaccinated so as to prevent nosocomial transmission of varicella.

The limitation of our study is that not all HCWs participated in our study, as 244 HCWs refused screening of serum VZV IgG, which may in turn lead to selection bias. However, further statistical analysis did not reveal any statistical differences by chi-square test between these HCWs and those who participated. Furthermore, this present study included a large number of HCWs with a wide age range and diverse occupations, therefore it still could provide a convincing approximation of the situation of VZV immunity in HCWs in China.

With the advancement in the field of transplantation and medicine, we are encountering more immunocompromised patients in our daily practice. Our study clearly demonstrated that assuming seropositivity based on self-reported history of varicella is unreliable, and significant proportion (10.6%) of existing HCWs are still susceptible to VZV. Further intervention should be implemented in the health care system to prevent hospital transmission of varicella. It is time for us to implement routine serum VZV IgG testing and immunization of HCWs if serum VZV IgG result turns out to be negative, not only to reduce the cost of outbreak investigation, but more importantly to ensure safety of both HCWs and our patients. Such intervention should not only be performed on newly recruited members, but also to existing healthcare workers in the hospital.

## Conclusions

Nosocomial transmission of VZV occurred repeatedly in the hospital setting. An alarming 11.6% of HCWs were seronegative for VZV, which might increase the risk of nosocomial infection and outbreak for other susceptible co-workers and patients. This is especially important in the setting of a teaching hospital where many immunocompromised patients were managed. Furthermore, the positive predictive value of self-reported varicella on seroprevalence rate in our study was lower than those reported in other countries, therefore serological testing of VZV antibodies with subsequent vaccination for all non-immune HCWs should be considered.

## Data Availability

The datasets used and analysed during the current study available from the corresponding author on reasonable request.

## Abbreviations

CDC: Centers for Disease Control and Prevention
ELISA: Enzyme-linked immunosorbent assay
FAMA: Fluorescent antibody to membrane antigen
HCWs: Healthcare workers
NPV: Negative predictive value
PCR: Polymerase Chain Reaction
PEP: Post-exposure prophylaxis
PPV: Positive predictive value
VZV: Varicella zoster virus
